# Phase 1b study of pembrolizumab (MK-3475; anti-PD-1 monoclonal antibody) in Japanese patients with advanced melanoma (KEYNOTE-041)

**DOI:** 10.1007/s00280-016-3237-x

**Published:** 2017-03-11

**Authors:** Naoya Yamazaki, Tatsuya Takenouchi, Manabu Fujimoto, Hironobu Ihn, Hiroshi Uchi, Takashi Inozume, Yoshio Kiyohara, Hisashi Uhara, Kazuhiko Nakagawa, Hiroshi Furukawa, Hidefumi Wada, Kazuo Noguchi, Takashi Shimamoto, Kenji Yokota

**Affiliations:** 1 Department of Dermatologic Oncology, National Cancer Center Hospital, 5-1-1 Tsukiji, Chuo-ku, Tokyo, 104-0045 Japan; 2 Department of Dermatology, Niigata Cancer Center Hospital, Niigata, Japan; 3 Department of Dermatology, Faculty of Medicine, University of Tsukuba, Tsukuba, Japan; 4 Department of Dermatology and Plastic Surgery, Faculty of Life Sciences, Kumamoto University, Kumamoto, Japan; 5 Department of Dermatology, Kyushu University School of Medicine, Fukuoka, Japan; 6 Department of Dermatology, Faculty of Medicine, University of Yamanashi, Yamanashi, Japan; 7 Department of Dermatology, Shizuoka Cancer Center, Shizuoka, Japan; 8 Department of Dermatology, Sapporo Medical University School of Medicine, Sapporo, Japan; 9 Department of Medical Oncology, Kindai University Faculty of Medicine, Osaka-sayama, Japan; 10 Department of Plastic and Reconstructive Surgery, Graduate School of Medicine, Hokkaido University, Sapporo, Japan; 11 Department of Dermatology, Yokohama City University School of Medicine, Yokohama, Japan; 12 MSD K.K., Tokyo, Japan; 13 Department of Dermatology, Nagoya University Graduate School of Medicine, Nagoya, Japan

**Keywords:** Pembrolizumab, Anti-PD-1 therapy, Melanoma, Immunotherapy, Japanese patients

## Abstract

**Purpose:**

This phase I b study evaluated the safety and anti-tumor activity of pembrolizumab in Japanese patients with advanced melanoma.

**Methods:**

Pembrolizumab (2 mg/kg) was given every 3 weeks (Q3W) for up to 2 years or until confirmed progression or unacceptable toxicity. The tumor response was assessed as per the Response Evaluation Criteria in Solid Tumors version 1.1 (RECIST v1.1) by both investigator review and central review.

**Results:**

Forty-two patients with advanced melanoma received pembrolizumab. A primary cutaneous histology was observed in 34 patients (81.0%), while a primary mucosal histology was observed in 8 patients (19.0%). Thirty-four patients (81.0%) experienced treatment-related adverse events (AEs). The most common treatment-related AEs were pruritus, maculopapular rash, malaise, and hypothyroidism. Grade 3–5 treatment-related AEs occurred in 8 patients (19.0%). The only grade 3–5 treatment-related AE reported in at least two patients was anemia. There were two treatment-related deaths (unknown cause and cerebral hemorrhage). Among the 37 evaluable patients, the confirmed overall response rates (ORRs) determined by central review were 24.1% (95% CI 10.3–43.5) for cutaneous melanoma and 25.0% (95% CI 3.2–65.1) for mucosal melanoma. The responses were durable, and the median duration of response was not reached in either population. The median overall survival (OS) was not reached, with a 12-month OS of 82.7% for cutaneous melanoma and 51.4% for mucosal melanoma.

**Conclusion:**

The safety profile of pembrolizumab in Japanese patients was similar to that reported in the previous clinical studies. Pembrolizumab provided promising anti-tumor activity in Japanese patients with advanced melanoma.

## Introduction

The malignant cell in melanoma is the melanocyte. Because melanocytes are located in the basal layer of the epidermis, melanoma is most commonly seen on the skin. However, melanoma can also arise on mucosal surfaces, such as the oral cavity, the upper gastrointestinal mucosa, the genital mucosa, as well as the uveal tract of the eye and leptomeninges [1, 2]. Internationally, approximately 230,000 new cases of melanoma are diagnosed per year, with over 70% of these diagnosed in Australia, Europe, or North America [3, 4]. Melanoma is a rare disease in Asia. In Japan, approximately 1300 new cases of melanoma were diagnosed and more than 600 patents died of melanoma in 2012, and the incidence is increasing [3]. The most common subtype of cutaneous melanoma in Caucasians is superficial spreading melanoma (SSM), accounting for approximately 60–70% of all melanomas [1]. In contrast, the most common subtype of cutaneous melanoma in Asia is acral lentiginous melanoma (ALM), accounting for approximately 50% of all melanomas [5–7]. ALM is the rare subtype in Caucasians, comprising <5% of cutaneous melanoma [6, 7]. The prognosis of SSM is the most favorable among the cutaneous melanoma subtypes [5, 8]. Mucosal melanomas represent only about 1.3% of all melanomas. Unlike cutaneous melanomas, mucosal melanomas are found at similar frequencies among patients of Black, Hispanic, or Asian origin, compared with non-Hispanic Caucasians [2]. The prognosis of mucosal melanoma is poorer than those of ALM and non-ALM cutaneous melanoma [8, 9]. ALM and mucosal melanoma have distinct genetic characteristics, such as less common BRAF and RAS mutations and a higher frequency of KIT mutations (10–20% for ALM, 15–20% for mucosal), compared to cutaneous melanomas (2%) [10–13] and a lower somatic mutational burden than that for SSM [14].

Pembrolizumab (formerly known as MK-3475) is a potent, highly selective, IgG4-k humanized monoclonal antibody that prevents programmed death 1 (PD-1) from binding with two ligands, programmed death ligand 1 (PD-L1) and programmed death ligand 2 (PD-L2). This agent was generated by grafting the variable region sequences of a mouse antihuman PD-1 antibody onto a human IgG4-k isotype framework containing a stabilizing S228P mutation of the Fc region. The administration of pembrolizumab at a dose of 10 mg/kg every 3 weeks (Q3W), 10 mg/kg every 2 weeks (Q2W), or 2 mg/kg Q3W resulted in ORRs of 25–52% in an advanced melanoma expansion cohort of a phase 1 study (KEYNOTE-001) [15]. Pharmacokinetic and pharmacodynamic analyses showed that the lowest dose with the full potential for anti-tumor activity was 2 mg/kg Q3W [16]. In a randomized phase 2 study performed in patients with ipilimumab-refractory melanoma (KEYNOTE-002), compared with chemotherapy, pembrolizumab at a dose of 2 or 10 mg/kg Q3W was well tolerated and significantly improved progression-free survival (PFS), reducing the risk of death or disease progression by 43 and 50%, respectively [17]. In a randomized phase 3 study performed in patients with ipilimumab-naïve melanoma (KEYNOTE-006), pembrolizumab at a dose of 10 mg/kg Q3W or Q2W significantly improved the PFS and overall survival (OS), compared with ipilimumab, reducing the risk of death or disease progression by 42% and the risk of death by 31–37%, respectively, as well as being associated with fewer occurrences of high-grade toxicity [18]. Randomized clinical studies of pembrolizumab demonstrated that no clinically meaningful differences in the safety or efficacy of pembrolizumab were evident between 2 mg/kg Q3W and 10 mg/kg Q3W or between 10 mg/kg Q3W and 10 mg/kg Q2W [17–20]. Thus, the administration of pembrolizumab at a dose of 2 mg/kg Q3W is currently approved in the US and over 60 other countries for the treatment of patients with advanced melanoma (unresectable or metastatic).

The safety and pharmacokinetic profiles for the Japanese patients were similar to those previously reported for Caucasian patients in a phase 1 study examining the use of pembrolizumab in Japanese patients with advanced solid tumors (KEYNOTE-011) [21]. Therefore, in this study, the safety and anti-tumor activity of pembrolizumab at a dose of 2 mg/kg Q3W were investigated in Japanese patients with advanced melanoma.

## Materials and methods

### Patient eligibility

This study was conducted based on the Declaration of Helsinki and the Guidelines for the Clinical Evaluation Methods of Anti-Cancer Drugs in Japan (Japanese Ministry of Health, Labor, and Welfare notification, November 1, 2005). The study was approved by the institutional review board of each study site.

The main eligibility criteria were as follows: a histologically confirmed diagnosis of locally advanced (unresectable Stage III) or metastatic (Stage IV) melanoma not amenable to local therapy; 0–2 prior lines of therapy (excluding adjuvant or neoadjuvant therapy) for melanoma; a patient age of 20 years or older; at least one measurable lesion as defined by RECIST v1.1; an Eastern Cooperative Oncology Group (ECOG) performance status of 0 or 1; and adequate hematologic, hepatic, and renal functions. The exclusion criteria included the administration of chemotherapy, radiotherapy, or biological therapy during the 4 weeks prior to enrollment; the previous treatment with a PD-1, PD-L1, or cytotoxic T-lymphocyte-associated protein 4 inhibitor; untreated and/or unstable central nervous system metastasis; or the presence of autoimmune disease.

All the patients provided informed consent, and the study was conducted in accordance with current Good Clinical Practice standards. This study was registered at ClinicalTrials.gov as NCT02180061.

### Study design and evaluation

This study was an open-label, non-randomized, multi-center phase Ib study of pembrolizumab in Japanese patients with advanced melanoma conducted at 12 sites in Japan. Pembrolizumab at a dose of 2 mg/kg Q3W was administered intravenously during a 30-min period, and treatment was continued until disease progression, the onset of unacceptable side effects, an investigator’s decision to discontinue treatment, the withdrawal of patient consent, or 24 months of therapy. Patients with a confirmed complete response who had received pembrolizumab for at least 6 months were allowed to discontinue therapy after receiving at least two doses beyond the determination of a complete response. The primary objectives of this study were to determine the safety, tolerability, and overall response rate (ORR) per RECIST v1.1 in patients with advanced cutaneous melanoma. The secondary objectives included evaluations of the duration of response and PFS per RECIST v1.1 as well as the OS in patients with advanced cutaneous melanoma. As exploratory objectives, the efficacy (ORR, duration of response, and PFS) of the treatment per RECIST v1.1 and the OS were also evaluated in patients with advanced mucosal melanoma.

Patients were evaluated using radiographic imaging to assess the response to treatment every 6 weeks starting at 12 weeks and continuing until 48 weeks; after 48 weeks, the patients were evaluated every 12 weeks. Tumor response was assessed using RECIST v1.1 by both investigator review and central review. If clinically stable, patients with first radiologic evidence of disease progression per RECIST v1.1 were permitted to continue receiving pembrolizumab until a second scan performed ≥4 weeks thereafter confirmed progression. Adverse events (AEs) were monitored throughout the study and were graded for severity according to the guidelines outlined in the NCI Common Terminology Criteria for Adverse Events (CTCAE), version 4.0.

Participation in this study required that an archival tissue sample or a newly obtained biopsy of a tumor lesion not previously irradiated be submitted for an evaluation of PD-L1 expression, which was conducted at a central laboratory. The expression status of PD-L1 was evaluated using immunohistochemistry (IHC) and the 22C3 antibody. PD-L1 positivity was defined as membranous PD-L1 expression in ≥1% of the tumor cells and associated immune cells. Both PD-L1 positive and negative patients were enrolled in this trial, and the clinical activities were evaluated in both subsets as pre-defined subgroups.

### Statistical analysis

Patients with advanced cutaneous melanoma were included in the primary efficacy analysis population. A confirmed ORR per RECIST v1.1, as assessed by central review, in patients with advanced cutaneous melanoma was used as the primary endpoint for the efficacy assessment. The full analysis set (FAS) population was used to analyze the efficacy data in this study. The FAS population consisted of all allocated patients with measurable lesions at the baseline scan, as assessed by central review, who had received at least one dose of the study treatment. The 95% confidence interval and a one-sided *P* value for testing the null hypothesis (ORR = 10%) based on a binomial distribution were calculated for the response rate. With approximately 28 evaluable patients with advanced cutaneous melanoma, the study had an approximately 90% power to detect a 25% difference in ORR under the null hypothesis of ORR = 10% with a type I error rate of 2.5% if the true ORR was 35%. The PFS, OS, and duration of response were estimated using the Kaplan–Meier method. The ASaT population was used for the primary safety analysis in this study. The ASaT population consisted of all the allocated patients who had received at least one dose of pembrolizumab. Immune-related AEs that were previously identified as important risks associated with pembrolizumab use were collected as Adverse Events of Special Interest (AEOSI). The AEOSIs included pneumonitis, colitis, thyroid disorders, hepatitis, hypophysitis, type 1 diabetes mellitus, uveitis, myositis, severe skin reactions, pancreatitis, nephritis, Guillain–Barré syndrome, and infusion-related reactions.

## Results

### Patient characteristics

Between July 2014 and March 2015, a total of 42 Japanese patients with advanced melanoma were enrolled in this study at 12 study sites in Japan and were treated with pembrolizumab (2 mg/kg Q3W). At the time of data cutoff (August 20, 2015), the median duration of follow-up was 10.3 months (range 2.6–12.6 months). The baseline characteristics of the patients are summarized in Table 1. The patients had a median age of 65 years (range 39–89 years), 61.9% were male, 81.0% had an ECOG performance status of 0, and 59.6% had not received any prior systemic therapy for advanced melanoma. A primary cutaneous histology was observed in 34 patients (81.0%), while a primary mucosal histology was observed in 8 patients (19.0%). The most frequent subtypes of melanoma were acral lentiginous melanoma (ALM: 12/42, 28.6%) and nodular melanoma (10/42, 23.8%). The BRAF V600 mutation was observed in 16.7% of the patients, and 50% had PD-L1-positive tissue samples. The median duration of treatment was 212.5 days (range 1.0–385.0 days), and the median number of treatments was 11.0 (range, 1.0–19.0). At the time of the analysis, 21 patients had discontinued pembrolizumab treatment: 5 because of an AE, 3 because of clinical progression, and 13 because of disease progression.

**Table 1:** Baseline patient characteristics

Characteristics	All treated patients (*n* = 42)
Age, year, median (range)	65 (39–89)
Sex, *n* (%)	
Male	26 (61.9)
Female	16 (38.1)
ECOG performance status, *n* (%)	
0	34 (81.0)
1	8 (19.0)
Tumor types, *n* (%)	
Cutaneous melanoma	34 (81.0)
NM	10 (23.8)
SSM	7 (16.7)
LMM	1 (2.4)
ALM	12 (28.6)
NC	4 (9.5)
Mucosal melanoma	8 (19.0)
BRAF status, *n* (%)	
Mutant	7 (16.7)
Wild	33 (78.6)
Undetermined	2 (4.8)
LDH, *n* (%)	
Normal	41 (97.6)
Elevated	1 (2.4)
PD-L1 expression^a^, *n* (%)	
Positive	21 (50.0)
Negative	13 (31.0)
Undetermined	8 (19.0)
Prior systemic therapies, *n* (%)	
None	12 (28.6)
Adjuvant/neoadjuvant	13 (31.0)
1	13 (31.0)
2	4 (9.5)

*ECOG* Eastern Cooperative Oncology Group, *NM* nodular melanoma, *SSM* superficial spreading melanoma, *LMM* lentigo maligna melanoma, *ALM* acral lentiginous melanoma, *NC* not classified, *LDH* lactate dehydrogenase

^a^Defined as membranous PD-L1 expression in >1% of tumor cells and associated immune cells as assessed using IHC with the 22C3 antibody

### Safety

Forty-two patients were treated with pembrolizumab (2 mg/kg Q3W), and all the patients were included in the safety analysis. The treatment-related AEs reported for all treatment cycles are summarized in Table 2. Thirty-four patients (81.0%) had at least one treatment-related AE of any grade. The most common treatment-related AEs were pruritus (*n* = 6, 14.3%), maculopapular rash (n = 6, 14.3%), malaise (*n* = 5, 11.9%), hypothyroidism (*n* = 4, 9.5%), vitiligo (*n* = 3, 7.1%), diarrhea (*n* = 3, 7.1%), an elevated AST level (*n* = 3, 7.1%), and an elevated eosinophil count (*n* = 3, 7.1%). Grade 3–5 treatment-related AEs occurred in 8 patients (19.0%). The only grade 3–5 treatment-related AE that was reported in at least 2 patients was anemia (4.8%). Two deaths (4.8%, unknown cause and cerebral hemorrhage) occurred and were considered by the investigator to have been related to the pembrolizumab treatment. Other than the above-mentioned 2 fatalities, 3 patients discontinued the pembrolizumab treatment because of a treatment-related AE (grade 2 arthralgia and grade 2 myalgia in one patient, grade 2 pneumonitis and grade 2 lung infection in one patient, and grade 2 hypophysitis in one patient). AEs of special interest based on a likely autoimmune or immune-related mechanism (irrespective of whether they were attributed to pembrolizumab by the investigator) occurred in 13 (31.0%) of the 42 patients. These events were hypothyroidism (*n* = 5, 11.9%), hypophysitis (*n* = 3, 7.1%), hyperthyroidism (*n* = 2, 4.8%), colitis (*n* = 2, 4.8%), infusion reaction (*n* = 2, 4.8%), pneumonitis (*n* = 1, 2.4%), severe skin reaction (*n* = 1, 2.4%), and uveitis (*n* = 1, 2.4%). The AEs of special interest with a severity of grade 3–5 were grade 3 colitis and grade 3 hypophysitis (*n* = 1 each).

**Table 2:** Treatment-related adverse events occurring in two or more patients with advanced melanoma after treatment with pembrolizumab

Adverse events	All treated patients (*n* = 42)	
	Any grade	Grade 3–5^a^
Skin and subcutaneous tissue disorders		
Pruritus	6 (14.3)	0
Maculopapular rash	6 (14.3)	0
Vitiligo	3 (7.1)	0
Skin hypopigmentation	2 (4.8)	0
Dry skin	2 (4.8)	0
General disorders and administration site conditions		
Malaise	5 (11.9)	0
Endocrine disorders		
Hypothyroidism	4 (9.5)	0
Hyperthyroidism	2 (4.8)	0
Hypophysitis	2 (4.8)	1 (2.4)
Gastrointestinal disorders		
Diarrhea	3 (7.1)	0
Nausea	2 (4.8)	0
Abdominal pain	2 (4.8)	0
Upper abdominal pain	2 (4.8)	0
Colitis	2 (4.8)	1 (2.4)
Laboratory results		
Elevated AST	3 (7.1)	0
Elevated eosinophil count	3 (7.1)	0
Elevated ALT	2 (4.8)	0
Blood and lymphatic system disorders		
Anemia	2 (4.8)	2 (4.8)

*AST* aspartate aminotransferase, *ALT* alanine aminotransferase

^a^Other grade 3–5 treatment-related AEs were grade 5 cerebral hemorrhage, grade 5 death from unknown cause, grade 4 hyperglycemia, grade 3 lymphopenia, grade 3 bile duct obstruction, grade 3 encephalopathy, and grade 3 drug eruption (*n* = 1 each)

### Efficacy

**Fig. 1:**
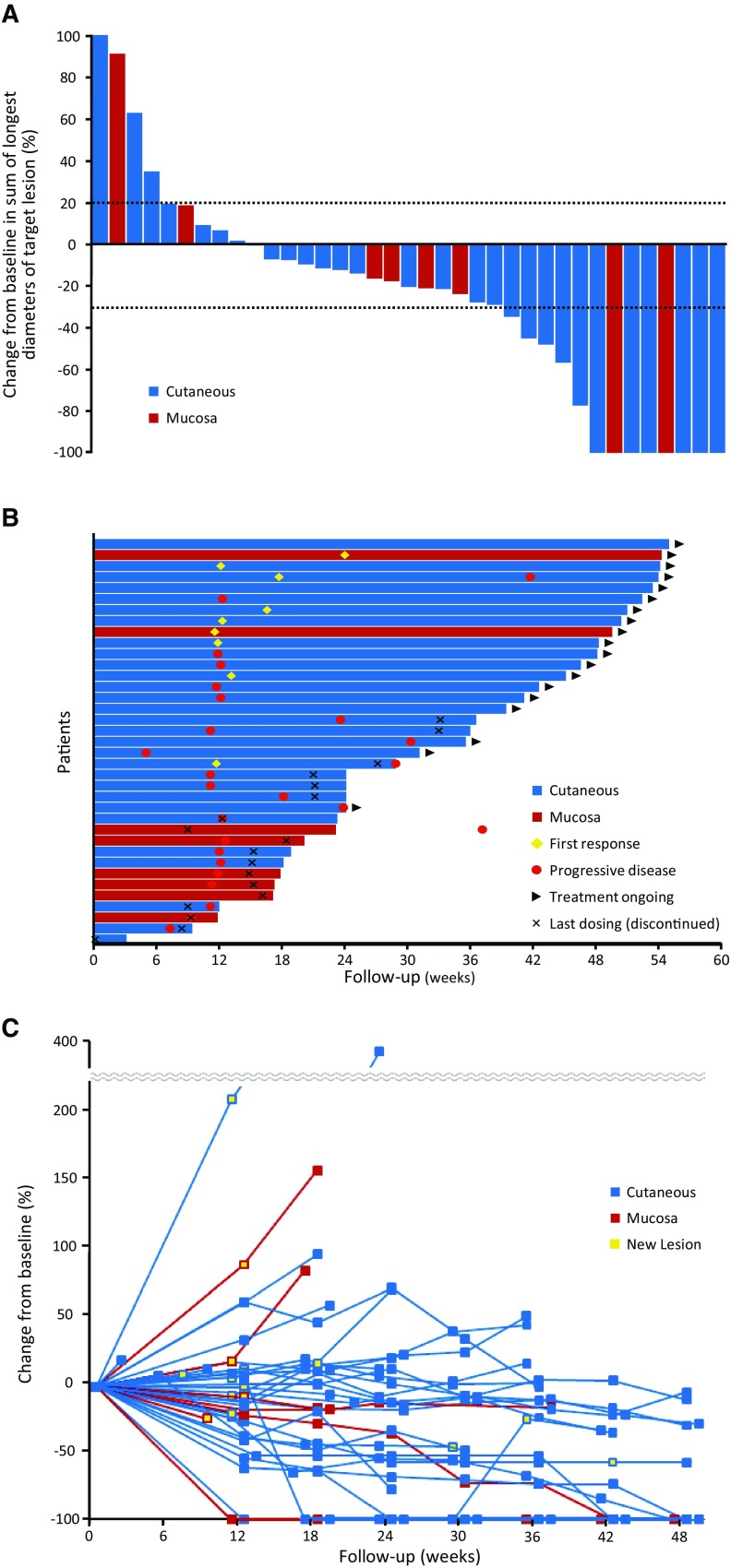
Anti-tumor activity of pembrolizumab per RECIST v1.1 by central review. **a** Best change from baseline in the sum of the longest target lesion diameters for each patient. **b** Treatment exposure and duration of response per patient. **c** Longitudinal changes in the sum of the longest target lesion diameters for each patient

**Fig. 2:**
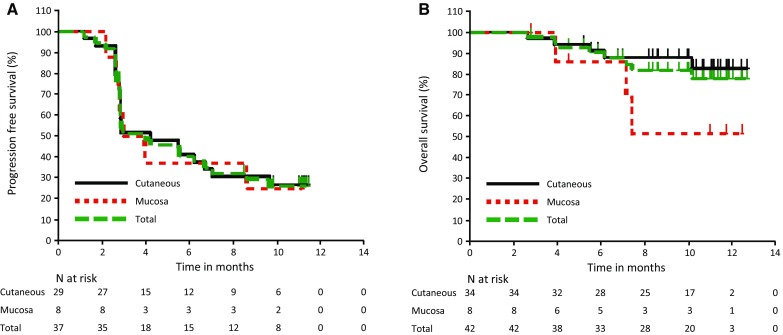
Kaplan–Meier analysis of **a** progression-free survival per RECIST v1.1 by central review and **b** overall survival

Among the 42 enrolled patients, 37 were evaluable for the tumor response performed by central review. Five patients without measurable lesions at baseline according to central review were excluded from the analysis. Of the 37 patients, 29 had cutaneous melanoma (primary efficacy evaluation population) and 8 had mucosal melanoma. The tumor response results per RECIST v1.1 are summarized in Table 3 and Fig. 1. A waterfall plot shows that 75.7% (28/37) of the patients experienced some degree of tumor shrinkage. The confirmed ORR, as assessed by central review, was 24.1% for the patients with cutaneous melanoma (7/29; 95% CI 10.3–43.5), and the lower limit of the 95% CI exceeded the 10% threshold response rate (*P* = 0.0216). The median time to response was 12.3 weeks among the patients with cutaneous melanoma. The responses were durable, and the median DOR was not reached; 5 (71.4%) of the 7 responders with cutaneous melanoma remained progression-free. The confirmed ORR, as assessed by central review, was 25.0% for the patients with mucosal melanoma (2/8; 95% CI 3.2–65.1). The median time to response was 17.8 weeks among the patients with mucosal melanoma. The median DOR was not reached, and 2 (100%) of the 2 responders with mucosal melanoma remained progression-free. Similar tumor response results were obtained by investigator review (Table 3). Kaplan–Meier curves of PFS per RECIST v1.1 by central review and OS are shown in Fig. 2. The median PFS as assessed by central review was 4.2 months (95% CI 2.8–7.0) for the patients with cutaneous melanoma, with a 6-month PFS rate of 41.4%. The median PFS was 3.4 months (95% CI 2.1–NA) for the patients with mucosal melanoma, with a 6-month PFS rate of 37.5%. The median OS was not reached in either population, with a 12-month OS rate of 82.7% for the patients with cutaneous melanoma and 51.4% for the patients with mucosal melanoma.

**Table 3:** Tumor response in patients with advanced melanoma treated with pembrolizumab

	Cutaneous melanoma *n* (%; 95% CI)	Mucosal melanoma *n* (%; 95% CI)	Total *n* (%; 95% CI)
Central review	(*n* = 29)	(*n* = 8)	(*n* = 37)
Overall response	7 (24.1%, 10.3–43.5)	2 (25.0%, 3.2–65.1)	9 (24.3%, 11.8–41.2)
Complete response	2 (6.9%, 0.8–22.8)	0	2 (5.4%, 0.7–18.2)
Partial response	5 (17.2%, 5.8–35.8)	2 (25.0%, 3.2–65.1)	7 (18.9%, 8.0–35.2)
Stable disease	7 (24.1%, 10.3–43.5)	2 (25.0%, 3.2–65.1)	9 (24.3%, 11.8–41.2)
Progressive disease	14 (48.3%, 29.4–67.5)	4 (50.0%, 15.7–84.3)	18 (48.6%, 31.9–65.6)
Non-evaluable	1 (3.4%, 0.1–17.8)	0	1(2.7%, 0.1–14.2)
Investigator review	(*n* = 34)	(*n* = 8)	(*n* = 42)
Overall response	9 (26.5%, 12.9–44.4)	3 (37.5%, 8.5–75.5)	12 (28.6%, 15.7–44.6)
Complete response	2 (5.9%, 0.7–19.7)	0	2 (4.8%, 0.6–16.2)
Partial response	7 (20.6%, 8.7–37.9)	3 (37.5%, 8.5–75.5)	10 (23.8%, 12.1–39.5)
Stable disease	14 (41.2%, 24.6–59.3)	2 (25.0%, 3.2–65.1)	16 (38.1%, 23.6–54.4)
Progressive disease	11 (32.4%, 17.4–50.5)	3 (37.5%, 8.5–75.5)	14 (33.3%, 19.6–49.5)
Median time to response, weeks (range)	12.3 (12–18)	17.8 (12–24)	12.3 (12–24)
Median duration of response, weeks (range)	NR (17 to 37+)	NR (24+ to 36+)	NR (17 to 37+)

*NR* not reached

Tumor tissues from 42 patients were available for use in a PD-L1 expression immunohistochemistry assay. PD-L1 positivity was defined as membranous PD-L1 expression in ≥1% of the tumor cells and associated immune cells as assessed using IHC with the 22C3 antibody. PD-L1 expression was positive in tumor samples from 21 patients (50.0%) and negative in tumor samples from 13 patients (31.0%). The PD-L1 status was not determined in tumor samples from eight patients (19.0%): seven patients had excessive melanin pigment in their tumor tissues, and one did not have a tumor with a defined border. The confirmed ORR as assessed by central review for both cutaneous and mucosal melanoma was 16.7% (3/18; 95% CI 3.6–41.4) for the patients with PD-L1-positive tumors and 36.4% (4/11; 95% CI 10.9–69.2) for the patients with PD-L1-negative tumors. The median PFS was 2.8 months, with a 6-month PFS rate of 27.8%, for the PD-L1 positive subgroup, while the median PFS was 5.4 months, with a 6-month PFS rate of 45.5%, for the PD-L1-negative subgroup. The OS was favorable for the PD-L1-positive subgroup (1-year OS rate: 83.3%), compared with that for the PD-L1-negative subgroup (1-year OS rate: 72.5%).

## Discussion

The primary objective of this study was to investigate the safety of single-agent pembrolizumab and the tumor response in Japanese patients with advanced melanoma. The dosing schedules in this study were 2 mg/kg Q3W, which is the approved dose for the treatment of advanced melanoma in the US and other countries. The safety profile of the Japanese patients with advanced melanoma was similar to that observed in clinical studies of pembrolizumab in non-Japanese patients [15–20]. The most common treatment-related AEs, which were reported in 3 patients or more, were pruritus, maculopapular rash, malaise, hypothyroidism, vitiligo, diarrhea, an elevated AST level, and an elevated eosinophil count. Grade 3–5 treatment-related AEs occurred in 19.0% of the patients. The only grade 3–5 treatment-related AE that was reported in at least 2 patients was anemia. Immune-related AEs occurred in 31.0% of the patients and included hypothyroidism, hypophysitis, colitis, hyperthyroidism, infusion reaction, pneumonitis, severe skin reaction, and uveitis. Immune-related AEs are distinct in both their mechanisms and management from AEs commonly associated with chemotherapy. The early recognition and treatment are essential to prevent morbidity and mortality, and adherence to established algorithms is recommended [22]. In this study, most immune-related AEs were of grade 1 or 2 and were manageable by interrupting the pembrolizumab treatment and/or providing medical intervention with steroid therapy and appropriate supportive care. At the time of the database cut-off date, one case of hypophysitis was not resolved; however, all the other events were alleviated or resolved, with the exception of events resulting in death.

In this study, the tumor response in patients with cutaneous melanoma was evaluated as the primary objective, and the tumor response in patients with mucosal melanoma was evaluated as an exploratory objective. The confirmed ORR as assessed by central review was 24.1% (7/29; 95% CI 10.3–43.5) for the patients with cutaneous melanoma, with the lower limit of the 95% CI exceeding the 10% threshold response rate (*P* = 0.0216), while the confirmed ORR was 25.0% (2/8; 95% CI 3.2–65.1) for the patients with mucosal melanoma. This study demonstrated the therapeutic benefits of pembrolizumab in Japanese patients with advanced cutaneous melanoma. The responses were durable, and the median DOR was not reached in either population. The study population of KEYNOTE-041 was ipilimumab-naïve, and 59.6% of the patients had not received any prior systemic therapy for advanced melanoma. Compared with the results from KEYNOTE-006, a randomized phase 3 trial comparing ipilimumab to pembrolizumab in non-Japanese patients with ipilimumab-naïve melanoma, the ORR for cutaneous melanoma was numerically lower than that (32.9–33.7%) observed in KEYNOTE-006, but the waterfall plots for both of the studies were similar. Pembrolizumab showed anti-tumor activity regardless of the PD-L1 expression status, though the sample sizes used for the subgroup analyses in this study were limited. Additional long-term OS analyses for KEYNOTE-041 were made with a data cut-off date of September 23, 2016 (median follow-up duration, 22. 0 months; range, 2.6–25.8 months). The median OS for the patients with cutaneous melanoma was 25.1 months (95% CI 15.8–25.1), with 12-month and 24-month OS rates of 76.1 and 57.0%, respectively. The median OS for the patients with mucosal melanoma was 23.2 months (95% CI 15.8–25.1), with 12-month and 24-month OS rates of 51.4 and of 25.7%, respectively. The OS results for cutaneous melanoma were similar to the final OS results for KEYNOTE-006 (12-month and 24-month OS rates of 68–74 and 55%, respectively) [23]. These OS results were consistent with those of the previous reports, which concluded that the prognosis of mucosal melanoma was poorer than that of cutaneous melanoma [8, 9].

The most frequent melanoma in this study was acral lentiginous melanoma (ALM, *n* = 12), followed by nodular melanoma (*n* = 10) and mucosal melanoma (*n* = 8). In this study, among the 12 patients with ALM, 2 had a CR and 1 had a PR, for an ORR of 25.0%. Recently, Shoushtari et al. reported that the ORR was 32.0% for patients with acral melanoma (*n* = 25) receiving pembrolizumab or nivolumab and 22.9% for those with mucosal melanoma (*n* = 35) [24]. On the other hand, the ORR for patients with metastatic uveal melanoma (*n* = 56) receiving pembrolizumab, nivolumab, or atezolizumab was reported to be 3.6% [25]. Patients with metastatic uveal melanoma were not enrolled in this study, because they were excluded according to the eligibility criteria. The anti-tumor activity of anti-PD-1 antibodies for advanced melanoma differed, depending on the subpopulation. Though the numbers of patients with ALM and mucosal melanoma were limited in this study, the ORR results were similar to those reported by Shoushtari et al. These results support the concept that pembrolizumab provides therapeutic benefits to certain subpopulations of patients, such as those with ALM or mucosal melanoma. Because of the rarity of mucosal melanoma, prospective clinical studies assessing the efficacy of systemic therapy have been rare, though a high, unmet medical need exists. Consequently, there are no standard guidelines for the treatment of mucosal melanoma at present. Therefore, to accelerate the development of treatment strategies for rare cancers, such as ALM and mucosal melanoma, a proactive approach to conducting clinical studies in countries/area with a relatively high incidence of the cancer is likely to become more and more important.

Recently, combination immunotherapy with ipilimumab and nivolumab received FDA and EMA approval. The combination of ipilimumab and nivolumab showed higher ORR of 57.6% as first-line treatment compared with each individual monotherapy. However, the combination induced 55.0% grade 3–4 treatment-related AEs and treatment discontinuation rates of 36.4% [26]. Therefore, a novel combination treatment with promising efficacy and less toxicity is still desired. The early clinical studies of pembrolizumab in combination with low-dose ipilimumab (1 mg/kg), talimogene laherparepvec (T-VEC), or epacadpstat, a selective oral inhibitor of indoleamine 2,3-dioxygenase 1(IDO1), showed acceptable safety profiles and promising efficacy in patients with advanced melanoma [27–29]. Phase 3 studies of pembrolizumab plus T-VEC, and pembrolizumab plus epacadostat are currently on-going in patients with advanced melanoma. Based on the positive results from this study, Japan is participating in the randomized phase 3 study of pembrolizumab versus placebo as adjuvant therapy after complete resection of high-risk stage III melanoma (EORTC1325/KEYNOTE-054), and will participate in phase 3 studies of combinations with pembrolizumab in advanced melanoma in the near future.

In conclusion, the safety profile of pembrolizumab in Japanese patients with advanced melanoma was similar to that reported in the previous studies. Pembrolizumab provided promising anti-tumor activity, supporting further clinical development of pembrolizumab for the treatment of Japanese patients with resectable stage III or advanced melanoma.

## References

[1] Kibbi N, Kluger H, Choi JN (2016). Melanoma: Clinical Presentations. Cancer Treat Res.

[2] Spencer HL, Mehnert JM (2016). Mucosal Melanoma: Epidemiology, Biology and Treatment. Cancer Treat Res.

[3] Ferlay J, Soerjomataram I, Ervik M, Dikshit R, Eser S, Mathers C, Rebelo M, Parkin DM, Forman D, Bray F. GLOBOCAN 2012 v1.0, Cancer Incidence and Mortality Worldwide: IARC CancerBase No. 11 10.1002/ijc.29210 25220842

[4] Jemal A, Bray F, Ferlay J, Ward E, Forman D, Center MM (2011). Global cancer statistics. CA Cancer J Clin.

[5] Ishihara K, Saida T, Otsuka F, Yamazaki N (2008). Statistical profiles of malignant melanoma and other skin cancers in Japan: 2007 update. Int J Clin Oncol.

[6] Chang JW, Yeh KY, Wang CH, Yang TS, Chiang HF, Wei FC (2004). Malignant melanoma in Taiwan: a prognostic study of 181 cases. Melanoma Res.

[7] Cress RD, Holly EA (1997). Incidence of cutaneous melanoma among non-Hispanic whites, Hispanics, Asians, and blacks: an analysis of California cancer registry data, 1988–93. Cancer Causes Control.

[8] Hao M, Zhao G, Du X, Yang Y, Yang J (2016). Clinical characteristics and prognostic indicators for metastatic melanoma: data from 446 patients in north China. Tumour Biol.

[9] Kuk D, Shoushtari AN, Barker CA, Panageas KS, Munhoz RR, Momtaz P (2016). Prognosis of mucosal, uveal, acral, nonacral cutaneous, and unknown primary melanoma from the time of first metastasis. Oncologist.

[10] Curtin JA, Busam K, Pinkel D, Bastian BC (2006). Somatic activation of KIT in distinct subtypes of melanoma. J Clin Oncol.

[11] Curtin JA, Fridlyand J, Kageshita T, Patel HN, Busam KJ, Kutzner H (2005). Distinct sets of genetic alterations in melanoma. N Engl J Med.

[12] Handolias D, Salemi R, Murray W, Tan A, Liu W, Viros A (2010). Mutations in KIT occur at low frequency in melanomas arising from anatomical sites associated with chronic and intermittent sun exposure. Pigm Cell. Melanoma Res.

[13] Kong Y, Si L, Zhu Y, Xu X, Corless CL, Flaherty KT (2011). Large-scale analysis of KIT aberrations in Chinese patients with melanoma. Clin Cancer Res.

[14] Furney SJ, Turajlic S, Stamp G, Thomas JM, Hayes A, Strauss D (2014). The mutational burden of acral melanoma revealed by whole-genome sequencing and comparative analysis. Pigment Cell. Melanoma Res.

[15] Hamid O, Robert C, Daud A, Hodi FS, Hwu WJ, Kefford R (2013). Safety and tumor responses with lambrolizumab (anti-PD-1) in melanoma. N Engl J Med.

[16] Patnaik A, Kang SP, Rasco D, Papadopoulos KP, Elassaiss-Schaap J, Beeram M (2105). Phase I study of pembrolizumab (MK-3475; anti-PD-1 monoclonal antibody) in patients with advanced solid tumors. Clin Cancer Res.

[17] Ribas A, Puzanov I, Dummer R, Schadendorf D, Hamid O, Robert C (2015). Pembrolizumab versus investigator-choice chemotherapy for ipilimumab-refractory melanoma (KEYNOTE-002): a randomised, controlled, phase 2 trial. Lancet Oncol.

[18] Robert C, Schachter J, Long GV, Arance A, Grob JJ, Mortier L (2015). Pembrolizumab versus ipilimumab in advanced melanoma. N Engl J Med.

[19] Robert C, Joshua AM, Weber JS, Ribas A, Hodi FS, Kefford RF et al (2014) Pembrolizumab (Pembro; MK-3475) for advanced melanoma (MEL): randomized comparison of two dosing schedules. Ann Oncol 25 (suppl 4). doi:10.1093/annonc/mdu438.42

[20] Robert C, Ribas A, Wolchok JD, Hodi FS, Hamid O, Kefford R (2014). Anti-programmed-death-receptor-1 treatment with pembrolizumab in ipilimumab-refractory advanced melanoma: a randomised dose-comparison cohort of a phase 1 trial. The Lancet.

[21] Shimizu T, Seto T, Hirai F, Takenoyama M, Nosaki K, Tsurutani J (2016). Phase 1 study of pembrolizumab (MK-3475; anti-PD-1 monoclonal antibody) in Japanese patients with advanced solid tumors. Invest New Drugs.

[22] Friedman CF, Proverbs-Singh TA, Postow MA (2016). Treatment of the immune-related adverse effects of immune checkpoint inhibitors: a review. JAMA Oncol.

[23] Schachter J, Ribas A, Long GV, Arance A, Grob JJ, Mortier L et al (2016) Pembrolizumab versus ipilimumab for advanced melanoma: Final overall survival analysis of KEYNOTE-006 [Abstract]. J Clin Oncol 34(suppl; abstr 9504)

[24] Shoushtari AN, Munhoz RR, Kuk D, Ott PA, Johnson DB, Tsai KK (2016). The efficacy of anti-PD-1 agents in acral and mucosal melanoma. Cancer.

[25] Algazi AP, Tsai KK, Shoushtari AN, Munhoz RR, Eroglu Z, Piulats JM et al (2016) Clinical outcomes in metastatic uveal melanoma treated with PD-1 and PD-L1 antibodies. doi:10.1002/cncr.30258 (Epub ahead of print) 10.1002/cncr.30258 PMC5767160 27533448

[26] Larkin J, Chiarion-Sileni V, Gonzalez R, Grob JJ, Cowey CL, Lao CD et al (2015) Combined nivolumab and ipilimumab or monotherapy in untreated melanoma. N Engl J Med 373:23e34. doi:10.1056/NEJMoa1504030. 10.1056/NEJMoa1504030 PMC5698905 26027431

[27] Long GV, Atkinson V, Cebon J, Jameson M, Fitzharris B, McNeil C et al (2016) Pembrolizumab (pembro) plus ipilimumab (ipi) for advanced melanoma: Results of the KEYNOTE-029 expansion cohort [Abstract]. J Clin Oncol 34(suppl; abstr 9506)

[28] Long GV, Dummer R, Ribas A, Puzanov I, VanderWalde A, Hans R et al (2016) Efficacy analysis of MASTERKEY-265 phase 1b study of talimogene laherparepvec (T-VEC) and pembrolizumab (pembro) for unresectable stage IIIB-IV melanoma [Abstract]. J Clin Oncol 34(suppl; abstr 9568)

[29] Gangadhar TC, Hamid O, Smith DC, Bauer TM, Wasser JS, Luke JJ (2015). Preliminary results from a Phase I/II study of epacadostat (incb024360) in combination with pembrolizumab in patients with selected advanced cancers. J Immuno Ther Cancer.

